# Supplementary material for: Predicting visual function by interpreting a neuronal wiring diagram
Source: Nature. 2024 Oct 2;634(8032):113–23. doi: 10.1038/s41586-024-07953-5 (PMC11446822; doi:10.1038/s41586-024-07953-5)
Supplement: Supplementary file 6 — CRF and ERF predictions for individual TmY4 and TmY9 cells. Analogous to Supplementary Data 3, but for TmY target types. Shown are the top four monosynaptic pathways, the strongest pathway passing through each of the top ten intermediary types (ranking from Extended Data Fig. 7), and the trisynaptic pathway Tm1–TmY–Dm3–TmY (see the section entitled Prediction of spatial normalization). [file 41586_2024_7953_MOESM6_ESM.zip › DataS4/TmY9q/720575940619167902.pdf]

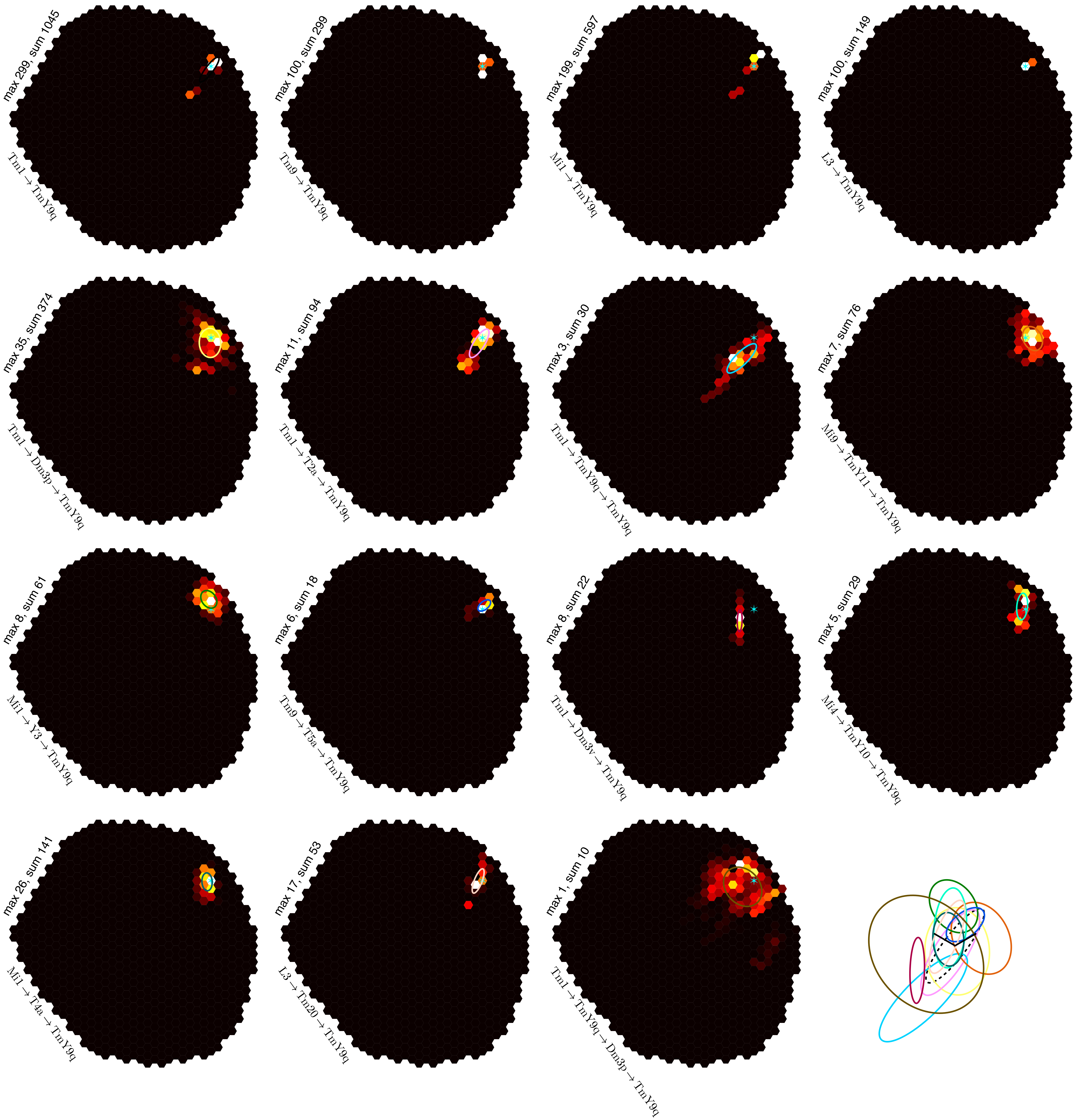
max 299, sum 1045
Tm1 → TmY9q
max 100, sum 299
Tm9 → TmY9q
max 199, sum 597
Mi1 → TmY9q
max 100, sum 149
L3 → TmY9q
max 35, sum 374
Tm1 → Dm3p → TmY9q
max 11, sum 94
Tm1 → T2a → TmY9q
max 3, sum 30
Tm1 → TmY9q → TmY9q
max 7, sum 76
Mi9 → TmY11 → TmY9q
max 8, sum 61
Mi1 → Y3 → TmY9q
max 6, sum 18
Tm9 → T5a → TmY9q
max 8, sum 22
Tm1 → Dm3v → TmY9q
max 5, sum 29
Mi4 → TmY10 → TmY9q
max 26, sum 141
Mi1 → T4a → TmY9q
max 17, sum 53
L3 → Tm20 → TmY9q
max 1, sum 10
Tm1 → TmY9q → Dm3p → TmY9q
